# Macromolecules Influence Cellular Competence and Expression Level of *IGFs* Genes in Bovine Oocytes In Vitro

**DOI:** 10.3390/ani12192604

**Published:** 2022-09-28

**Authors:** Uğur Şen, Emre Şirin, Hasan Önder, Selçuk Özyürek, Magdalena Kolenda, Beata Sitkowska

**Affiliations:** 1 Department of Agricultural Biotechnology, Ondokuz Mayis University, Samsun 55139, Turkey; 2 Department of Agricultural Biotechnology, Kırşehir Ahi Evran University, Kırşehir 40100, Turkey; 3 Department of Animal Science, Ondokuz Mayis University, Samsun 55139, Turkey; 4 Department of Veterinary, Erzincan Binali Yıldırım University, Erzincan 24500, Turkey; 5 Department of Animal Biotechnology and Genetic, Faculty of Animal Breeding and Biology, Bydgoszcz University of Science and Technology, 85-796 Bydgoszcz, Poland

**Keywords:** bovine, oocytes, maturation, macromolecules, *IGFs*, antioxidant activity, cellular development

## Abstract

**Simple Summary:**

The macromolecule content of culture media can affect the maturation competence of oocytes, which influences the subsequent in vitro development of embryos. This study was designed to determine the effects of macromolecules on cellular competence and the transcript level of insulin-like growth factors (*IGF1*, *IGF2*) and their receptors in bovine oocytes. The current study showed that bovine serum albumin (BSA) and fetal calf serum (FCS) improved nuclear maturation and protein biosynthesis (especially FCS). Polyvinyl alcohol did not support the antioxidant defense mechanism due to decreased glutathione peroxidase enzyme activity. The expression of the *IGF1* gene could not be detected in all experimental groups, but BSA and FCS increased the transcript level of the *IGF2* gene. Moreover, oocyte maturation with BSA increased the transcript level of the *IGF1R* gene, whereas the transcript level of the *IGF2R* gene was similar among macromolecule supplementation groups. The BSA and FCS could improve in vitro bovine oocyte development due to supporting cellular characteristics.

**Abstract:**

In vitro maturation (IVM) of mammalian oocytes, which influences subsequent in vitro development of embryos, is affected by the macromolecule content in culture media for the success of oocyte maturation competence, in which the cytoplasmic and nuclear reprogramming events occur. The insulin-like growth factor family (*IGFs*) promotes the maturation of bovine oocytes and the expansion of cumulus cells and also inhibits apoptosis. This study was, therefore, designed to examine the effects of macromolecules (bovine serum albumin, BSA; fetal calf serum, FCS; and polyvinyl alcohol, PVA) on in vitro nuclear maturation, total cellular protein, glutathione peroxidase (GPx) enzyme activity, and the gene expression level of *IGF1*, *IGF2*, and their receptor in bovine oocytes. Oocytes obtained from bovine ovaries were cultured in bicarbonate-buffered medium 199 supplemented with 4 mg/mL BSA, 10% FCS, 1 mg/mL PVA, and without macromolecule supplement (control) during 22 h in the air with a humidified atmosphere and 5% CO_2_ at 38.5 °C temperature. Supplementation of BSA and FCS increased (χ^2^ = 9.84; *p* < 0.05) the percentages of oocytes that reached metaphase II compared to the control and PVA. The amount of protein per ml of cell extracts of oocytes matured in FCS supplemented culture media was higher (*p* < 0.05) than the oocytes in the PVA and control. The levels of GPx enzyme activity in cell extracts isolated from oocytes in each experimental group did not change over time, but the GPx enzyme activity in oocytes matured in PVA-supplemented culture media was lower (*p* < 0.05) than in oocytes in the other experimental groups. Transcript for the *IGF1* gene was not detected in all experimental groups, but the supplementation of BSA and FCS significantly elevated the transcript level of the *IGF2* gene. In addition, the maturation of oocytes with BSA-supplemented media increased the transcript level of the *IGF1R* gene, whereas the transcript level of the *IGF2R* gene was similar among macromolecule supplementation groups. The current study concluded that BSA and FCS could improve in vitro bovine oocyte development due to supporting nuclear maturation and increasing the total cellular protein content, GPx enzyme, and transcript activity.

## 1. Introduction

The in vitro production (IVP) of livestock embryos allows for greater reproductive use of genetically superior animals and improves animal husbandry by reducing intermittent production [1]. The IVP of bovine embryos is widely used for commercial purposes and scientific research, but current in vitro embryo production biotechnologies still lack adequate the mimicry of in vivo conditions [2,3,4]. The high mortality ratio of in vitro produced embryos is a significant problem facing the cattle breeding industry, which compromises reproductive efficiency, genetic improvement, and the development and use of biotechnology related embryo production [5]. To deal with this problem, it was necessary to determine the effects of the culture media ingredients used for in vitro oocyte maturation, which affects subsequent embryonic development, on the molecular and cellular competence of oocytes, such as polypeptide growth factor expression, cellular protein synthesis, and antioxidant activity.

It has been shown that the kinetics of in vitro-produced bovine embryos can be affected by serum supplementation during in vitro maturation (IVM), in vitro fertilization (IVF), and embryo cultures [6]. Most of the previous studies indicated that the presence of fetal calf serum (FCS) during IVM increases the maturation rate of oocytes and subsequent embryonic development in bovine. In addition, FCS has proven to be a superior protein supplement compared to bovine serum albumin (BSA) [7]. In the absence of serum during IVM or IVF, the first and fourth cell cycles in early embryos are prolonged by 4–5 h [6]. Despite multiple reports of the beneficial effects of serum supplementation during IVM on the developmental potential of mammalian oocytes, some controversial reports indicated the detrimental effects of serum supplementation during IVM in some animal species [8,9,10]. Serum supplementation may alter the metabolic activity and expression of some imprinted genes of oocytes, resulting in lower blastocyst yields and pregnancy rates and postnatal abnormalities, such as large offspring syndrome [11,12]. In addition, the expression level of some gene transcripts, such as DNA methyltransferase 3a, heat shock protein 70, desmosomal glycoprotein desmocollin III, interferon tau, and insulin-like growth factors 1 and 2 (*IGF1* and *IGF2*), and their receptors was altered according to different culture serum component [13].

The macromolecule content of the maturation medium has an important effect on oocyte competence, as reflected by morula and blastocyst yield after IVF. Many studies involving bovine embryo IVP have achieved better results when using macromolecules of animal origin [1,10,14]. Despite their undefined, complex, and variable nature, the most common animal-derived macromolecule supplements are FCS and BSA in bovine oocyte maturation media. The BSA and FCS can significantly affect the synthesis and storage of specific mRNA and protein forms, which are essential for the developmental competence of oocyte maturation in vitro and further embryonic development [13]. Knowing the protein content of oocytes, which is an indicator of the cellular development process, is essential for interpreting the information obtained from studies on parameters such as energy metabolism and protein synthesis, which are involved in controlling oocytes and embryo development [15]. While information about the protein content in preimplantation embryos of mice, rats, rabbits, pigs, and bovine is available in previous studies [16], there is a lack of such information on bovine oocytes.

Studies of cellular and molecular factors operating during oocyte maturation and fertilization provide the basis for identifying conditions for the IVP of embryos and their possible applications in basic research and animal breeding [17]. Bradshaw and Rubin [18] reported that polypeptide growth factors may play a pivotal role in the maturation of mammalian oocytes and stimulate an increase in cell size and/or cell number during preimplantation embryo development. Insulin-like growth factors, which increase cell proliferation and mitogenesis and regulate apoptosis, have essential effects on ovarian physiology, follicular development, oocyte maturation, and embryonic growth and development in mammals [19]. Animal-derived proteins (BSA and fetal serum) are the most frequently used media supplements and have hormones, growth factors, vitamins, and numerous other unknown factors, but their use is still controversial. Therefore, to solve this uncertainty, chemically defined culture media supplemented with synthetic macromolecules (polyvinyl alcohol and polyvinyl pyrrolidone) is of great interest. Wrenzycki et al. [20] noticed that the culture medium supplementation of different protein sources or macromolecules affects the expression of developmentally important genes in embryos. However, the possible effects of animal-derived proteins and macromolecule supplementation to maturation media on expression levels of cell proliferation, differentiation, survival, growth, apoptosis, and regeneration-related genes and their receptors (*IGF1*, *IGF1R*, *IGF2*, *IGF2R*) and the underlying mechanisms have not yet been established.

As a result of intracellular metabolic activity, reactive oxygen species (ROS) are produced as a by-product, mainly including oxidative phosphorylation [21,22]. The ROS are unstable atoms due to lacking electrons. Although ROS, which is produced in limited amounts in response to physiological stimuli, is considered to mediate cellular signal transduction processes involved in the growth and protection of granulosa cells from apoptosis, as well as some essential functions of reproduction such as the fertilization ability of spermatozoa, the sperm capacitation process and acrosome reaction, and the generation of excess results in oxidative stress. The primary role of ROS is highly associated with cellular damages such as the inactivation of cellular enzymes, membrane lipid peroxidation, and DNA lesions and strand breaks [23,24]. Different complementary mechanisms are involved in the protection of gametes from ROS. The free radicals scavengers, which are found in follicular and oviduct fluids, protect oocytes and embryos against oxidative stress in vivo [25,26]. However, in the application of in vitro oocyte maturation, free oxygen radicals can be produced during the isolation of the oocyte from the follicle environment [22]. To protect the oocyte and embryo from the adverse effects of ROS, in vitro oocyte maturation and embryo production systems were developed in culture supplementations to scavenge ROS, including enzymatic and non-enzymatic antioxidant agents [27]. Animal-derived macromolecules supply important components, such as nutrients, pH buffers, and antioxidants [28,29]. Although the effects of FCS and BSA supplementation on oocyte maturation competence and the early development of mammalian IVP embryos have been reported, to our knowledge, limited information has been published regarding the relationship between animal-derived macromolecules and oxidative stress during the IVM of bovine oocytes.

Conventionally animal derived macromolecules are required for the IVM of oocytes to improve the in vitro production of embryos. The possible effects of macromolecule supplementation to in vitro bovine oocyte maturation culture media on cellular development, protein accumulation, antioxidant enzyme systems, apoptosis, cell proliferation, and survival-related genes’ expression levels have not been determined, or there is limited information. Therefore, this study aimed to determine the effects of BSA, FCS, and PVA, supplemented with in vitro bovine oocyte maturation culture, on nuclear maturation, total cellular protein, GPx enzyme activity, and the relative expression level of cell proliferation, differentiation, survival, growth, apoptosis, and regeneration-related genes and their receptors (*IGF1*, *IGF1R*, *IGF2*, *IGF2R*).

## 2. Materials and Methods

All chemicals and commercial animal cell culture media used in the experiments were purchased from Sigma-Aldrich, Turkey; different ones were also indicated.

### 2.1. Isolation of Cumulus-Oocyte Complexes

Bovine ovaries were obtained immediately after slaughter from a local slaughterhouse. Ovaries were transported to the laboratory in 0.9% NaCl (S5886; *w/v*) containing 0.1% *v/v* antibiotic solution (A5955; 10,000 IU penicillin, 10 mg streptomycin, and 25 μg amphotericin B per mL) at 35 °C within a few hours (~2 to 3 h) of slaughter. Cumulus-oocyte complexes (COCs) were aspirated from follicles with diameters of 2–8 mm using a 10 mL disposable syringe attached to an 18-gauge needle. The COCs were washed several times in Hepes-modified medium 199 (M7528) and supplemented with 1% *v/v* antibiotic-antimycotic solution and 100 μg/mL L-glutamine. The COCs were examined morphologically, and only COCs with non-atretic cumulus investment and compact and evenly granulated cytoplasm were chosen for IVM.

### 2.2. Experimental Design and In Vitro Oocyte Maturation

The IVM media were prepared with commercial medium 199-buffered sodium bicarbonate (M4530) containing Earle’s salts and L-glutamine-supplemented 5.5 μg/mL sodium pyruvate and 1% *v/v* antibiotic-antimycotic solution. The selected COCs were washed three times in Hepes-modified medium 199 and were randomly subjected to the following medias: (a) IVM + 4 mg/mL BSA (fatty acid free) (n = 434), (b) IVM + 10% FCS (n = 426), (c) IVM + 1 mg/mL PVA (n = 540), and (d) IVM without a macromolecule supplement (n = 525). After washing in the free supplemented IVM, approximately 25 to 35 COCs were transferred to 500 μL IVM of each experimental group in each well of the 4-well multi dishes (Nunc, Roskilde, Denmark) covered with 300 μL of mineral oil. The COCs were matured for 22 h in the air with a humidified atmosphere and 5% CO_2_ at a 38.5 °C temperature.

### 2.3. Maturation Characteristics of Oocytes

**Figure 1:**
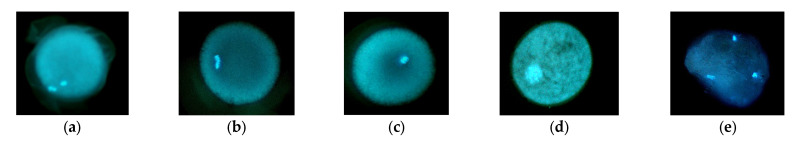
Nuclear status of bovine oocytes. Stained with Hoechst 33,342: (**a**) M-II, (**b**) T-I, (**c**) M-I, (**d**) GVBD, and (**e**) DG (400×).

Cumulus cell expansion rates of oocytes in each experimental group were examined at the end of the maturation culture using a stereomicroscope. Oocytes, which exhibited a cumulus cell expansion, were considered maturated. After cumulus cell evaluation, oocytes were washed several times in Hepes-modified medium 199. Some of the oocytes in each experimental group (BSA, n = 152; FCS, n = 153; PVA, n = 153; and control, n = 152) were completely denuded of cumulus cells by vortexing immediately after washing. Denuded oocytes were treated with an acetic acid: ethanol (1:3, *v/v*) mixture for 24 h at 4 °C for the fixation of meiosis status. The fixed oocytes were stained with 90% glycerol (diluted in dPBS; pH 7.4) containing 10% (*w/v*) Hoechst 33342 (H-6024) at approximately 23 °C room temperature for 15 min. The meiosis status evaluations of oocytes were examined under the fluorescent microscope through a 420-nm barrier filter. The meiosis status of oocytes was classified (Figure 1), without knowing the experimental groups, by an independent researcher as metaphase II (M-II), telophase I (T-I; together with anaphase I), metaphase I (M-I), germinal vesicle breakdown (GVBD), and degenerated (DG; together with chromosome aberrations).

### 2.4. Protein Extraction

In vitro matured and denuded bovine oocytes in each experimental group were washed three times in Ca^2+^ and Mg^2+^ free PBS and were stored in 0.5 mL of sterile Eppendorf tubes (approximately 25 / 10 μL) at −80 °C until protein isolation. The total protein isolation of oocytes in each experimental group was performed by the radio immunoprecipitation assay (RIPA) buffer method with some modifications [30]. In this method, 100 denuded oocytes in each experimental group were thawed at room temperature, and a mixture with 100 µL of RIPA buffer (50 mM sodium chloride, 1% Triton X-100, 0.1% sodium dodecyl sulfate; 50 mM Tris 8, pH: 8.0) and 1 mL of protease inhibitor cocktail was put in their storage tubes. Thhis mixture was then homogenized on ice using a probe-sonicator (Vibra-Cell model VCX 130; Sonics and Materials Inc., Newtown, CT, USA) with a tip diameter of 3 mm at 75% amplitude for 1 min, 2 times, with 30-s intervals. The samples were stored at 4 °C for 1 h for protein solubilization and centrifuged at 19,000× *g* at 4 °C for 10 min. After centrifugation, the supernatant was removed, and the pellet bottom of the tube was washed with 200 μ of ethanol (95%) and centrifuged at 19,000× *g* at 4 °C for 10 min. The amount of protein in the pellet was determined by the Bradford Coomassie brilliant blue method (Bradford, 1976) using a UV spectrophotometer at a wavelength of 590 nm. The protein content was calculated using a standard curve prepared based on bovine serum albumin. The analysis for each sample was duplicated, and mean values were calculated from three replicates.

### 2.5. Glutathione Peroxidase (GPx) Enzyme Activity

In vitro matured and denuded bovine oocytes (100 oocytes in each experimental group) were frozen and thawed two times and homogenized on ice using a probe-sonicator with a tip diameter of 3 mm at 75% amplitude for 1 min, 2 times, with 30-s intervals. The samples were then centrifuged at 10,000× *g* at 4 °C for 20 min, and supernatants were separated. The GPx enzyme activity in the supernatants for each experimental group was determined by the colorimetric method using a commercial GPx Assay kit (Northwest Life Science Specialties, LLC, Vancouver, Washington, DC, USA) as suggested by the producer (Paglia and Valentine, 1967). The GPx enzyme activity was observed 7 times for 3 min at 30 s intervals at a wavelength of 340 nm using a UV spectrophotometer (Shimadzu UV-1800), and the analysis for each experimental group was duplicated.

### 2.6. Total RNA Isolation, Synthesis of cDNA, and qRT-PCR Analyses

In vitro matured and denuded 100 oocytes in each experimental group were transferred to 0.5 mL sterile tubes and submitted for total RNA extraction. A commercial RNA kit (NucleoSpin^®^ RNA kit) was used for RNA isolation, and the manufacturer protocol was followed for the whole process. Genomic DNA was eliminated by digestion with DNase I (Thermo Fisher Scientific Inc., Waltham, MA, USA). The quality and quantity of isolated RNA were evaluated by the A260/A280 ratio using a NanoDrop™ 2000/2000c spectrophotometer (Thermo Fisher Scientific Inc., Waltham, MA, USA), and all RNA samples showed A260/A280 values within the range of 2.01 to 2.08 and A260/ A230 values above 2. RNA was resuspended in 10 mL of elution buffer and stored at −80 °C until use.

Isolated RNA was converted to cDNA using a commercial cDNA kit (BIORAD iScript cDNA, 1708890) in the Thermal Cycler (BIORAD) device. Primers used for the amplification of genes were designed using online tools (https://www.ncbi.nlm.nih.gov/tools/primer-blast/) (accessed on 4 March 2021) based on the related gene sequences of bovine (Table 1). A quantitative real-time polymerase chain reaction (qRT-PCR) assay was performed with the CFX96 Touch Real-Time PCR Detection System (Bio-Rad Laboratories, Hercules, CA, USA) using a commercial qPCR kit (5× HOT FIREPol EvaGreen qPCR Mix Plus, Solis BioDyne, Tartu, Estonya). Reactions were carried out in 50 µL volumes consisting of 10 µL of cDNA. Reactions were optimized to provide the maximum amplification efficiency for the *IGFs* and their receptor genes (which was >90%). The thermal profile consisted of an initial activation 12 min melting step at 95 °C followed by 40 cycles at 95 °C for 15 s (denaturation), 60 °C for 20 s (annealing), and 72 °C for 20 s (elongation). β-Actin was selected as the housekeeping gene to normalize the expression of the *IGFs* and their receptor genes. Three independent biological replicates of each treatment group were performed. A hypothetical control group was created to determine *IGFs* and their receptor gene expression levels using the lowest Ct value of β-Actin and the highest Ct value of the target gene in bovine oocytes cultured in different maturation media. The relative expression levels of the genes were calculated by the 2^−ΔΔCt^ method.

### 2.7. Statistical Analysis

The chi-square (χ^2^) test was used to determine the effects of macromolecules on the maturation parameters of bovine oocytes. The data on gene expression level, protein content, and GPx enzyme activity were analyzed using a completely randomized design by the General Linear Model (GLM) procedure of the SPSS package program. Duncan’s test was used for tested significant differences between means, and results were computed as mean ± standard error of the mean. Statistical significance was considered at *p* < 0.05.

## 3. Results

The rates of cumulus cell expansion and the meiosis status of bovine oocytes are presented in Table 2. Oocytes with exhibited full cumulus expansion and in the M-II stage of meiosis were considered mature in all experimental groups. The supplementation of FCS and BSA to the culture media increased (χ^2^ = 9.56; *p* < 0.05) the expansion rate of cumulus cells by approximately 24% compared to the PVA and control groups at the end of the maturation culture period. Similarly, the supplementation of FCS and BSA was found to increase the percentage of oocytes that reached M-II at the end of the maturation period (χ^2^ = 9.84; *p* < 0.05). However, there were no significant differences among experimental groups in terms of the percentage of oocytes reaching the T-I stage. The percentage of oocytes that reached M-I (χ^2^ = 10.25; *p* < 0.05) and degenerated (χ^2^ = 10.96; *p* < 0.05) was statistically lower in FCS and BSA groups than those of PVA and the control.

The total protein content in bovine oocytes is presented in Table 3. In the study, it was determined that the protein amount in ml of cell extracts isolated from oocyte-matured FCS supplemented in the culture media was higher than those of the PVA and control groups (*p* < 0.05). Similarly, the amount of protein per oocyte was higher in oocytes matured in media supplemented with FCS than in the PVA and control (*p* < 0.05).

**Figure 2:**
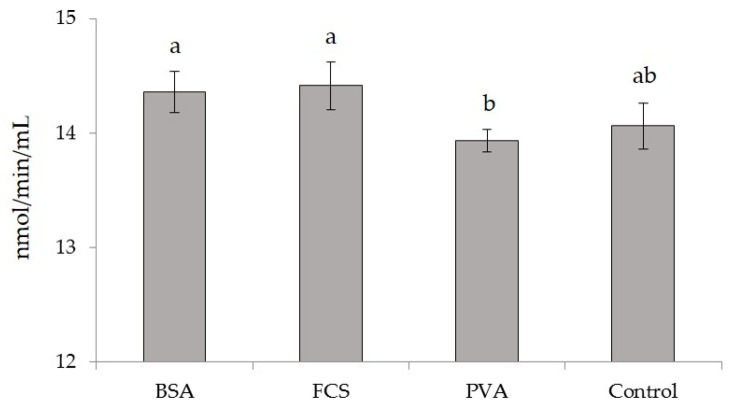
The mean glutathione peroxidase (GPx) enzyme activities (nmol/min/mL) in bovine oocytes matured in media supplemented with different macromolecules. BSA = bovine serum albumin, FCS = fetal calf serum, PVA = polyvinyl alcohol. The error bars represent the standard error of means, and bars with different letters are significantly different at *p* < 0.05.

Mean GPx enzyme activities (nmol/min/mL) in bovine oocytes matured in media supplemented with BSA, FCS, and PVA at different measurement times are presented in Table 4. It was determined that the levels of GPx enzyme activity in the cell extracts isolated from the oocytes in each experimental group did not change over time, but the mean GPx enzyme activity of oocytes in the PVA group was lower (*p* < 0.05) than those of the BSA and FCS groups (Figure 2).

**Figure 3:**
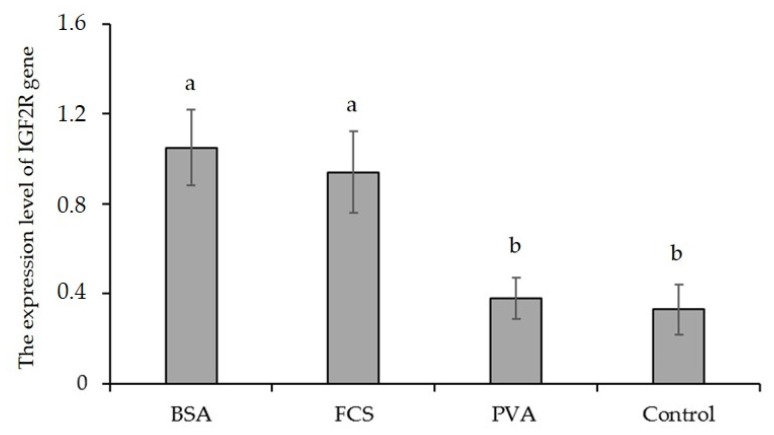
The expression level of the *IGF2* gene in bovine oocytes matured in media supplemented with different macromolecules. BSA = bovine serum albumin, FCS = fetal calf serum, PVA = polyvinyl alcohol. The error bars represent the standard error of means, and bars with different letters are significantly different at *p* < 0.05.

**Figure 4:**
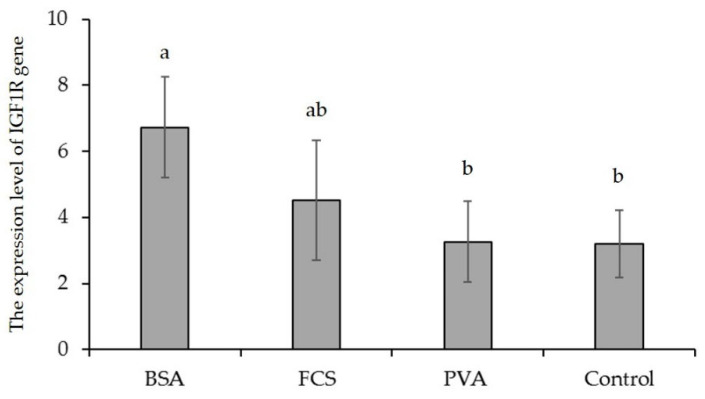
The expression levels of the *IGF1R* gene in bovine oocytes matured in media supplemented with different macromolecules. BSA = bovine serum albumin, FCS = fetal calf serum, PVA = polyvinylyl alcohol. The error bars represent the standard error of means, and bars with different letters are significantly different at *p* < 0.05.

**Figure 5:**
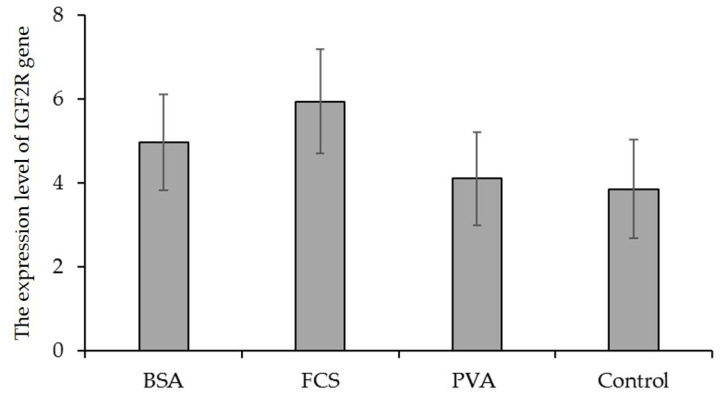
The expression level of the *IGF2R* gene in bovine oocytes matured in media supplemented with different macromolecules. BSA = bovine serum albumin, FCS = fetal calf serum, PVA = polyvinyl alcohol. The error bars represent the standard error of means.

The cDNA samples of experimental groups were amplified with β-actin primers. The transcript level of the housekeeping gene was found to be constant in each experimental group (data not shown). The transcript for the *IGF1* gene was not detected in any of the investigated oocyte samples from experimental groups as a result of the qRT-PCR assay, but BSA and FCS increased the transcript level of the *IGF2* gene. Considering the macromolecule supplementation of IVM for oocyte IVM, significant differences in the relative expression of *IGF2* and *IGF1R* genes between oocytes in each experimental group were noticed, whereas the transcript level of the *IGF2R* gene was similar among macromolecule supplementation groups (Figure 3, Figure 4 and Figure 5). According to the expression value, *IGF2* and *IGF1R* gene expression increased in all experimental groups. However, the supplementation of BSA and FCS significantly increased the transcript level of the *IGF2* and *IGF1R* genes. The expression level of the *IGF2* gene in BSA- and FCS-supplemented bovine oocytes was ~0.7-fold higher than those of the PVA and control. Similarly, BSA-supplemented bovine oocytes had ~2.1-fold higher expression levels of the *IGF1R* gene compared to the PVA and control.

## 4. Discussion

The dynamics of nuclear maturation in oocytes can be used as an indicator of subsequent embryonic developmental competence after fertilization, and it is affected by the macromolecule ingredient of IVM culture media [29]. During the nuclear maturation process of oocytes, the nucleus progresses from the germinal vesicle to the M-II stage. To evaluate the effects of nuclear maturation on subsequent embryonic development, oocytes with first polar body emission at the correct time, which is an indicator of nuclear maturation, have a higher developmental potential from the zygote to the blastocyst stage after fertilization [31]. In the present study, we observed a higher cumulus cell expansion and M-II stage, which means an earlier first PB emission, in oocytes matured in media supplemented with BSA and FCS compared to the PVA and control. The study results show that the supplementation of PVA as a defined macromolecule to the maturation medium did not improve the rate of reaching the M-II stage of meiosis, an indicator of nuclear maturation in bovine oocytes. In support of this finding, the previous studies confirm that the blastocyst rate of embryos obtained from matured oocytes in a culture medium supplemented with PVA was significantly reduced [32,33]. In contrast to our result, Eckert and Niemann [34] showed that nuclear maturation rates in estrous cow serum, BSA, and PVA groups did not differ significantly. In contrast, Mingoti et al. [29] reported that macromolecular and serum supplements did not affect the ratio of oocytes reaching the M-II stage of meiosis at the 20% O_2_ and the 24 h maturation culture time. Results of the current study indicated that BSA and FCS accelerated the dynamics of the nuclear maturation of oocytes, which may suggest that oocytes can reach the blastocyst stage at a higher rate after fertilization. However, previous studies reported that the supplementation of FCS to the maturation medium increased the cumulus expansion and nuclear maturation of bovine oocytes more than the BSA supplementation [7,35,36]. All these phenomena indicate that future research should study the effects of macromolecular and serum supplements on the nuclear and cytoplasmic maturation of bovine oocytes more extensively.

It has been reported that the progression of mammalian oocytes from the prophase to M-II stages of meiosis is highly dependent on the synthesis of new proteins rather than follicle size [37]. The reorganization of protein synthesis following germinal vesicle breakdown is crucial to completing the nuclear maturation of oocytes [38]. During the oocyte maturation process, many specific proteins are synthesized such as ribosomal, mitochondrial and zona pellucida proteins, glycoproteins, histones, tubulin, actin calmodulin, lactate dehydrogenase, creatine kinase, and glucose-6-phosphate dehydrogenase [38]. These specific proteins and enzymes are necessary for pronuclear formation during different stages of meiosis. Therefore, the structural proteins and enzymes are constantly synthesized and stored throughout oocyte growth and maturation [39]. In the present study, there were important differences in oocytes in terms of protein content among experimental groups; a marked increase in the protein content of oocytes matured in FCS-supplemented culture media compared with the PVA (~1.68 fold) and control (~1.80 fold) was observed. The lower protein content of oocytes matured in PVA-supplemented media and control media might represent depletion, or, on the contrary, the higher protein content in oocytes with FCS supplementation might represent protein accumulation, which is involved in the successful cellular development process and the control of subsequent embryo development [15,16]. The average protein content (0.102 μg) of pooled bovine oocytes from each group was higher compared with that reported for goat (57.1 ng) [40] and mouse (16.5 ng) [41]. However, it is similar to the results of previous studies on bovine (0.126 μg) [16], buffalo (0.16 μg), and sheep (0.12 μg) [38].

Oxidative stress interferes with spermatozoa and oocyte viability and embryo development and health in mammals [42]. A series of antioxidant enzymes and nonenzymatic processes protect gametes and embryos against ROS damage during oocyte maturation and the early development stage of the embryo [22,43]. The in vitro environment favors the increase in ROS concentration, given the small volume compared to the in vivo system. Therefore, in vitro systems have developed mechanisms to control ROS levels, including enzymatic (superoxide dismutase, glutathione peroxidase, and catalase) and non-enzymatic (α-tocopherol, ascorbic acid, β-carotene, and glutathione) antioxidant agents [27]. Previous studies reported that an enzymatic antioxidant defense system, which includes specific enzymes or their transcripts, was identified in follicular fluid [22,25], oocytes [44,45], and the oviduct [26]. Indeed, the enzymatic antioxidant defense system against hydrogen peroxide (H_2_O_2_), which is a major contributor to oxidative damage, includes the GPx enzyme and classic catalase. GPx enzyme activity is one of the most important components of cellular antioxidant activity for the protection of membranes, proteins, and DNA against damage by lipid peroxidation [22,23]. GPx is an intracellular antioxidant enzyme that catalyzes the reduction of H_2_O_2_ to water and oxygen, as well as catalyzing the reduction of peroxide radicals to alcohols and oxygen to limit its harmful effects [25,26]. In addition, the GPx enzyme uses H_2_O_2_ in the reaction where reduced glutathione is converted to oxidized glutathione, thus preventing the accumulation of H_2_O_2_ in the cell [25,26]. In the present study, the effect of macromolecular and serum supplements on glutathione peroxidase enzyme activity, which prevents intracellular accumulation of H_2_O_2_, was investigated. The present study argued that the supplementation of different macromolecules into in vitro bovine oocyte maturation media altered the GPx enzyme activity of oocytes. The GPx enzyme activity of oocytes matured in PVA-supplemented media was lower than those of BSA and FCS oocytes. Yanar et al. [46] suggested that the low activity of GPx enzymes can increase the amount of reduced glutathione, whereas a decrease in glutathione reductase activity can depress the amount of reduced glutathione. The mutual activities of these enzymes may have an effect on the reduced glutathione concentration in the cells and may allow a rise in ROS concentrations and oxidative stress. FCS is the liquid fraction of clotted blood from fetal calves, depleted of cells, fibrin, and clotting factors, but containing many nutritional and macromolecular elements essential for cell growth [47]. Moreover, BSA is a significant component of FCS. BSA acts primarily as a carrier protein for hormones, fatty acids, trace minerals, vitamins, and iron. However, it also plays a role in stabilizing extracellular fluid volume and maintaining osmotic balance, as well as binding harmful toxins and free radicals [48]. FCS and BSA may have contributed to the harvest of ROS because of the components it contains. Therefore, the result of the present study indicated that PVA might less support the family of the GPx enzymes than BSA and FCS.

The expression levels of genes encoding various growth factors are considered an indicator of developmental competence and viability in mammalian oocytes and embryos [21,49]. The *IGFs* (*IGF1* and *IGF2*) have various anabolic effects on cell metabolism, including the stimulation of amino acid and glucose transport, protein and nucleic acid synthesis, and also cell proliferation and differentiation [49,50,51]. Wrenzycki et al. [20] reported that in vitro culture conditions and, consequently, oocyte and embryo quality significantly affect the transcript levels of developmentally important genes. Although the relative expression of the *IGF1* and *IGF2* genes and their receptors in bovine oocytes and embryos, which were cultured in vitro, has been well documented, this issue should be carefully re-examined, taking into account the in vitro culture conditions and media ingredients. Previous studies reported that types 1 and 2 of *IGFs* and their receptors are expressed in bovine oocytes and embryos [17,52,53]. The present study examined *IGF1*, *IGF2*, and their receptor gene’s relative expressions in bovine oocytes, which were matured in different BSA-, FCA-, or PVA-supplemented culture media. We could not determine the relative gene expression levels because the transcript level of the *IGF1* gene could not be detected in all experimental groups as a result of the qRT-PCR assay. However, oocyte maturation with BSA significantly elevated the transcript level of the *IGF1R* gene. Similarly, the transcript level of the *IGF2* gene was significantly altered in matured oocytes and was determined to be highest in the supplementation of BSA and FCS. These observations are in agreement with the results of Warzych et al. [49], who reported that the transcript for the *IGF1* gene was not detected in any IVM medium supplement groups, whereas the presence of BSA increased the transcript level of the *IGF1R* and *IGF2* genes. Moreover, Wang et al. [21] reported that, although *IGF1R*, *IGF2*, and *IGF2R* mRNA were detected in matured bovine oocytes in vitro, the transcript for the *IGF1* gene could not be detected in matured bovine oocytes. The answer to this inconsistency regarding *IGF1* expression in bovine oocytes may lay in the detection method applied (primer design, PCR amplification). A sensitive, quantitative, real-time PCR system for *IGF1* gene expression in vitro maturated bovine oocytes has not been defined; thus, some other still not identified factors may be responsible for these differences. Lonergan et al. [52] used RT-nested PCR, whereas the other two groups used the conventional RT-PCR technique. Moreover, specific components used in the experiments, particularly differences in the vitro oocytes culture environment, may influence *IGF1* gene expression in bovine oocytes. Lonergan et al. [52] and Niemann and Wrenzycki [53] reported that expression levels of developmentally necessary genes are affected by the in vitro culture conditions or media ingredients.

Previous studies reported that the expression of *IGF1R*, *IGF2*, and *IGF2R* genes in immature or in vitro matured oocytes in bovine was observed [17,20,51,52]. In this study, the supplementation of macromolecules to maturation media had no significant influence on the relative expression level of *IGF2R* genes in matured oocytes. BSA or FCS supplementation elevated only the relative expression level of the *IGF2* gene. Wrenzycki et al. [20] suggested that the transcript abundance of the *IGF2* gene may be a potential marker of embryonic viability in vitro. Results of the present study indicate that BSA and FCS may be the optimal supplements to maturation medium under the described conditions due to the higher transcript level of the *IGF2* gene in matured oocytes. However, it is rather tricky to unequivocally conclude that PVA exerts the most detrimental effect on oocytes since BSA and FCS supplementation did not alter the transcript level of the *IGF2R* gene.

## 5. Conclusions

In conclusion, the results of this study suggest that BSA and FCS supplementation, despite their undefined, complex, and variable nature, to bovine IVM medium is the best option in the described experimental design to improve cellular characteristics and elevate the relative expression level of *IGF* genes. In contrast, PVA supplementation did not improve the in vitro cellular and molecular adequacy of bovine oocytes. In addition, the results of the present study suggest that PVA might less support the intracellular enzymatic antioxidant system than BSA and FCS.

The maturation of oocytes is the most sensitive developmental stage to environmental insults; thus, the causes of abnormalities occurring in later embryonic developmental stages, such as the blastocyst, can be attributed to stress factors. Embryonic developmental competence following fertilization may be regulated by small changes in the expression level of several genes already in oocytes. The biological functions of *IGFs* might be performed by various growth factors such as GF, EGF, TGF, etc. Therefore, in the future, in vitro studies should consider the effects of macromolecule supplementations on the complex system of *IGFs* and cellular activity in bovine oocytes to increase the success rate of in vitro bovine embryo production.

## Figures and Tables

**Table 1:** Details of primer pairs used for bovine oocyte RT-PCR reactions.

Genes	Primer sequence (5′-3′)	AT (°C)	FS (bp)	GBAN
*IGF1*	F-CATTCATTCAGCAGGCTTGTCTAA R-TGATGGAGAAGGGAGTGGGATA	59	129	X15726
*IGF1R*	F-CAACTGTCCTGACATGCTGTTTGAGC R-CCGCCTCCATCTCGTCCTTGAC	64	113	X54980
*IGF2*	F-TCTACTTCAGCCGACCATCCA R-TTCGGAAGCAACACTCTTCCA	60	72	X53553
*IGF2R*	F-AGTGTGTGTGACTTCGTGTTTGAG R-TGGAGAGGCTGGACAGGTTG	61	124	X54980
β-actin	F-CGTGAGAAGATGACCCAGATCA R-GGGACAGCACAGCCTGGAT	59	79	U39357

AT = annealing temperature, FS = fragment size, GBAN = gene bank accession number.

**Table 2:** Meiosis status of bovine oocytes matured in media supplemented with BSA, FCS, and PVA.

	CE		M-II		T-I		M-I		DG	
	n	%	n	%	n	%	n	%	n	%
BSA	302	90.96 ^a^	110	72.36 ^a^	22	14.47	13	8.55 ^a^	7	4.61 ^a^
FCS	302	93.50 ^a^	127	83.01 ^a^	13	8.50	9	5.88 ^a^	4	2.61 ^a^
PVA	302	68.95 ^b^	87	56.87 ^b^	16	10.46	32	20.92 ^b^	18	11.77 ^b^
Control	305	71.93 ^b^	74	48.68 ^b^	21	13.82	37	24.34 ^b^	20	13.15 ^b^

^a,b^ Different letters in the same column indicate significant difference (*p* < 0.05). BSA = bovine serum albumin, FCS = fetal calf serum, PVA = polyvinyl alcohol, CE = fully cumulus expansion, M-II = metaphase II, T-I = telophase I, M-I = metaphase I, DG = degenerate (including chromosome aberrations, germinal vesicle, and germinal vesicle break down).

**Table 3:** Total protein amounts in bovine oocytes matured in media supplemented with BSA, FCS, and PVA.

	BSA	FCS	PVA	Control
Protein (µg/mL)	2.435 ± 0.166 ^ab^	3.619 ± 0.351 ^a^	2.165 ± 0.362 ^b^	2.009 ± 0.383 ^b^
Protein (µg/oocyte)	0.098 ± 0.006 ^ab^	0.144 ± 0.014 ^a^	0.086 ± 0.014 ^b^	0.080 ± 0.015 ^b^

^a,b^ Different letters in the same column indicate significant differences (*p* < 0.05). BSA = bovine serum albumin, FCS = fetal calf serum, PVA = polyvinyl alcohol.

**Table 4:** The glutathione peroxidase (GPx) enzyme activities (nmol/min/mL) in bovine oocytes matured in media supplemented with BSA, FCS, and PVA at different measurement times.

Time (s)	BSA	FCS	PVA	Control
0	14.5 ± 0.13	14.6 ± 0.20	14.0 ± 0.19	14.3 ± 0.55
30	14.5 ± 0.09	14.5 ± 0.20	14.0 ± 0.18	14.2 ± 0.31
60	14.3 ± 0.09	14.4 ± 0.20	14.0 ± 0.09	14.2 ± 0.31
90	14.3 ± 0.08	14.4 ± 0.20	13.9 ± 0.19	14.1 ± 0.32
120	14.2 ± 0.07	14.4 ± 0.20	13.9 ± 0.19	14.1 ± 0.34
150	14.2 ± 0.07	14.3 ± 0.20	13.9 ± 0.20	14.0 ± 0.35
180	13.9 ± 0.19	14.3 ± 0.20	13.8 ± 0.18	13.9 ± 0.36

BSA = bovine serum albumin, FCS = fetal calf serum, PVA = polyvinyl alcohol.

## Data Availability

To obtain the data, please contact the corresponding author.
